# Potential Energy Surfaces and Quantum Yields for Photochromic Diarylethene Reactions

**DOI:** 10.3390/molecules18055091

**Published:** 2013-05-02

**Authors:** Shinichiro Nakamura, Kingo Uchida, Makoto Hatakeyama

**Affiliations:** 1 RIKEN Research Cluster for Innovation, Nakamura Laboratory, 2-1 Hirosawa Wako, Saitama 351-0198, Japan; 2 Department of Materials Chemistry, Faculty of Science and Technology, Ryukoku University, Seta, Otsu 520-2194, Japan

**Keywords:** diarylethene, photochromism, quantum yield, theoretical study

## Abstract

Photochromic diarylethenes (DAEs) are among the most promising molecular switching systems for future molecular electronics. Numerous derivatives have been synthesized recently, and experimental quantum yields (QYs) have been reported for two categories of them. Although the QY is one of the most important properties in various applications, it is also the most difficult property to predict before a molecule is actually synthesized. We have previously reported preliminary theoretical studies on what determines the QYs in both categories of DAE derivatives. Here, reflecting theoretical analyses of potential energy surfaces and recent experimental results, a rational explanation of the general guiding principle for QY design is presented for future molecular design.

## 1. Introduction

This review covers our theoretical study on the quantum yields (QYs) of the photochromic isomerizations of diarylethene (DAE). Photochromism is the reversible light-induced transformation of a compound between two forms having different absorption spectra [1,2]. The DAEs with heterocyclic aryl groups show photochromism and have attracted much attention owing to their potential applications as molecular sensors in optoelectronic and optobioelectronic devices [3,4]. For such applications, both the cyclization and cycloreversion reactions should have large QYs as well as large absorption coefficients.

Many groups have reported experimental research devoted to improving various critical properties of DAEs [3,4], and we have reported theoretical studies devoted to: (i) explaining the origin of thermal stability [5,6]; (ii) designing absorption wavelengths [7]; (iii) identifying the factors determining the quantum yield for photochromic isomerization [8]; (iv) evaluating a variety of experimental spectroscopic data (NMR [9,10], Raman [11], IR [12,13,14,15], and ESR [16,17]); (v) finding applications that utilize hole and electron transport properties [18]; (vi) designing the environmental field effect around the molecule (solvent [9]; polymer [19] and crystal [20]), and (vii) elucidating nonlinear response properties [21]. 

The QY for a molecule’s photochromic isomerization is the most difficult of all these properties to design because it is a counterintuitive one and its actual value must be measured after the molecule has been synthesized. Here we complete a theoretical discussion of QY that we have partially reported previously [8].

## 2. Two Categories of DAE Molecules

**Scheme 1:**
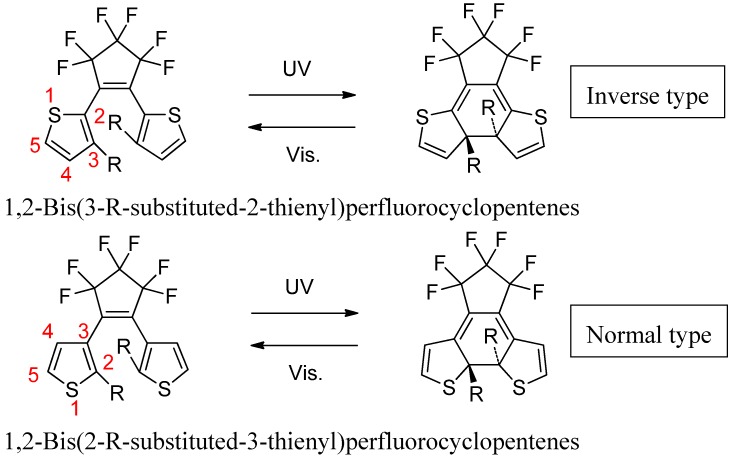
Two categories of DAE.

Amongst the many DAE derivatives there are two categories of molecules: one with 3-thienyl groups as the aryl groups (normal type) and the other with 2-thienyl groups as the aryl groups (inverse type) (Scheme 1). The DAEs with 3-thienyl groups have been well studied [1,2,3,4,22,23,24,25,26,27]. The photochromic properties of the DAEs with 2-thienyl aryl groups, however, are very different from those of the normal DAEs. The molecular structure of bis(2-thienyl)perfluorocyclopentenes, in which thiophene rings are linked at the 2-position to the ethane moiety is the reverse of that of the normal bis(3-thienyl)perfluorocyclopentenes. The DAE closed form is always more conjugated then the corresponding open form, as indicated by the redshift of the absorption peaks. Electrons in the open forms of normal type DAEs are more localized on the thiophene rings, while in the open form of the inverse type, are more delocalized on the molecular core [28].

The absorption wavelengths and quantum yields of the cyclization and cycloreversion reactions of the derivatives reflect these structural differences, because photochromic properties are affected by the substituents attached to the reactive carbon atoms. The substituent effects in bis(3-thienyl)-perfluorocyclopentene derivatives have been systematically studied [8,26], but those in bis(2-thienyl)perfluorocyclopentene derivatives have not been investigated as thoroughly, because the inverse type of derivatives were not so attractive for a long time to synthetic chemists, as they did not have a large variation of colours in their closed forms. The elucidation of both types of diarylethenes—normal and inverse types—is of critical importance for any future molecular electronics applications. 

## 3. DAE Photochromism Mechanism Inferred from the Potential Energy Surfaces

**Figure 1:**
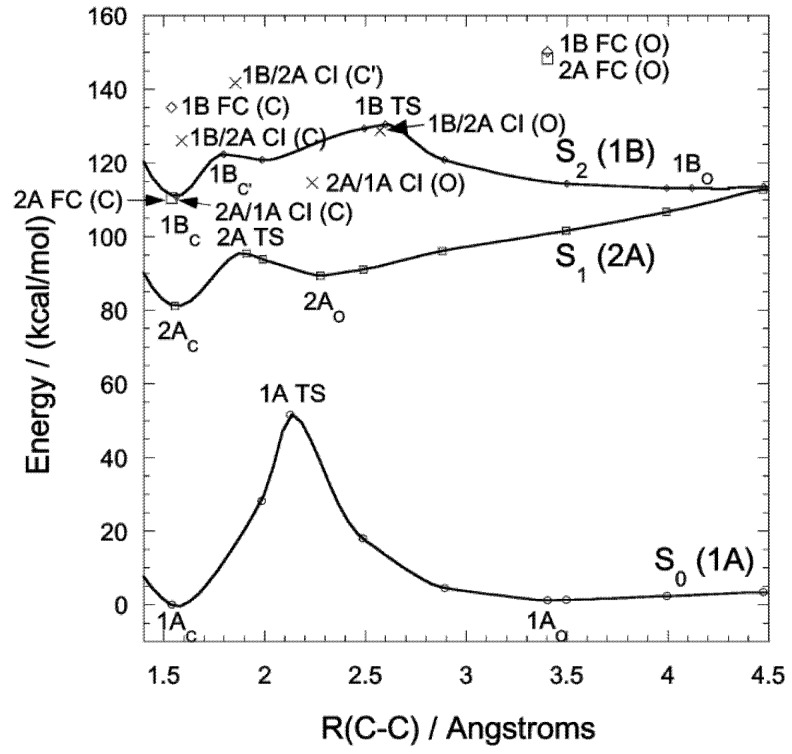
PES profiles for a normal-type model molecule. Each point was obtained, using the state-specific CASSCF(10,10) method with the 6-31G basis set, by fixing R(C–C) at different distances and optimizing other geometrical freedoms. Stationary points (1Ao, 1Ac, 2Ac, 2Ao, 1Bc and1Bo) and saddle points (1ATS, 2ATS and 1BTS) were obtained without geometry constraints at the C2 symmetry and were verified by frequency analysis. Conical Intersections (2A/1A CI(C), 2A/1A CI(O), 1B/2A(C), 1B/2A(C0), and 1B/2A(O) are obtained by the state-averaged CASSCF(10,10) method. FC denotes the Franck-Condon state. See ref. [31] for details.

**Scheme 2:**
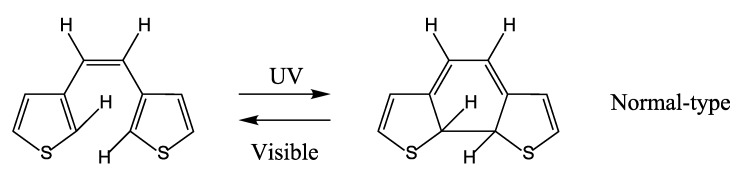
Normal-type model molecule.

Photochromic ring-closing (cyclization) and ring-opening (cycloreversion) reactions are explained at a fundamental level by the Woodward-Hoffman rules [29], based on which we analyzed the factors determining the thermal stability of the closed forms of DAEs and dinaphthylethene derivatives [5,6]. We then studied the details of the reaction mechanism by obtaining the potential energy surfaces (PESs) for the photochromic reactions of DAEs. Focusing on the model molecule shown below (Scheme 2), we obtained the PESs by calculation at the CASSCF level (Figure 1) [30,31], where the critical role of the conical intersections (CIs) is clarified. In fact, the location of the CIs on the PESs determines whether or not photochromic reactions occur and, if they do, determines the order of their QYs. 

**Scheme 3:**
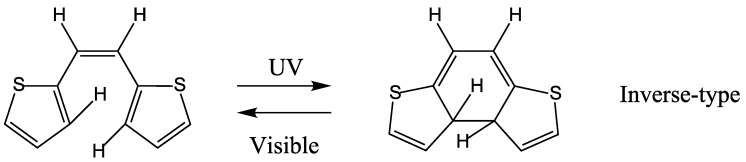
Inverse-type model molecule.

**Figure 2:**
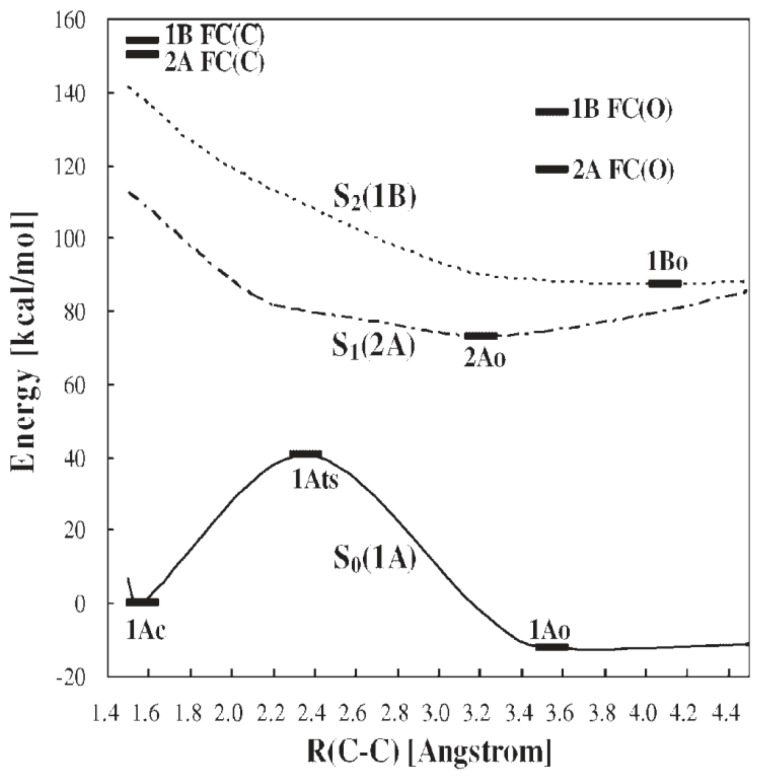
PES profiles for an inverse-type model molecule. The level of the calculation here was same as that for the normal-type molecule shown in Figure 1 (see ref. [8] for details).

The PES profiles obtained when the same level of the calculation was carried out for the inverse-type model molecule (Scheme 3) are shown in Figure 2.

Both the QYs for cyclization and cycloreversion reactions obviously need to be optimized for future applications. The cycloreversion reaction takes place by visible light irradiation, where the open form does not absorb light, and the process is a pure fading. To the contrary, in the UV region, both forms absorb the light, and direct and inverse processes may take place. Kobatake *et al.* reported an experimental comparative study of diarylethene crystals showing that the distance between the reactive carbons (C2 and C2'), R(C-C) should be less than 4Ǻ for cyclization to occur [32]. Then the cyclization QY can be 1.0 in the crystalline phase, probably because there is no room for side reactions (with low or no activation barrier) or nonradiative decay (within 10 ps) [33]. Morinaka *et al.* reported that the cyclization QY is also very high (0.81) in hexane solution [34]. It is naturally possible to explain these facts when we obtain the PES for cyclization [8]. Cycloreversion QYs, in contrast, especially those for normal-type DAEs, are rarely large. Hereafter, we will discuss experimentally obtained cycloreversion QYs in consideration of the cycloreversion PES. 

## 4. QYs for Normal-Type DAEs

**Figure 3:**
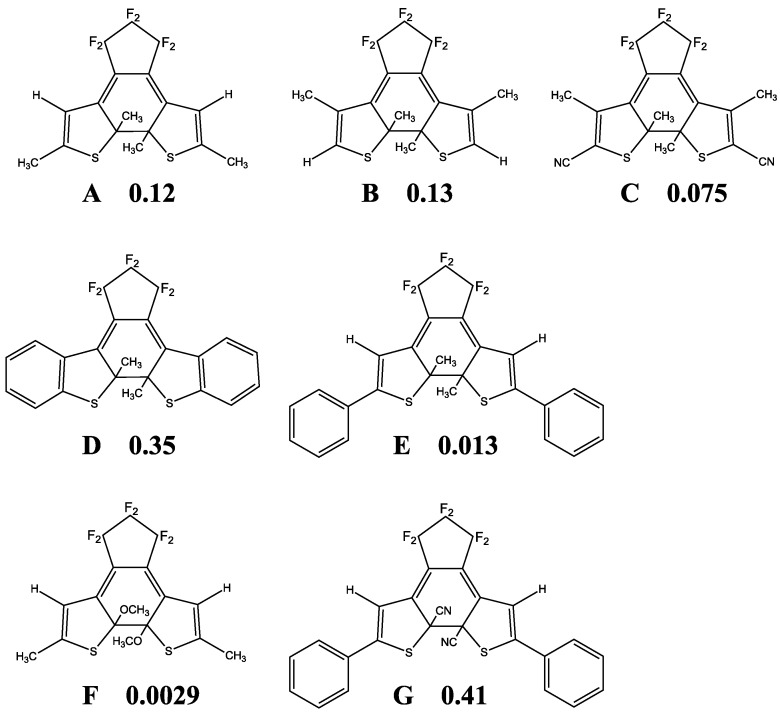
Experimental QYs for cycloreversion.

**Figure 4:**
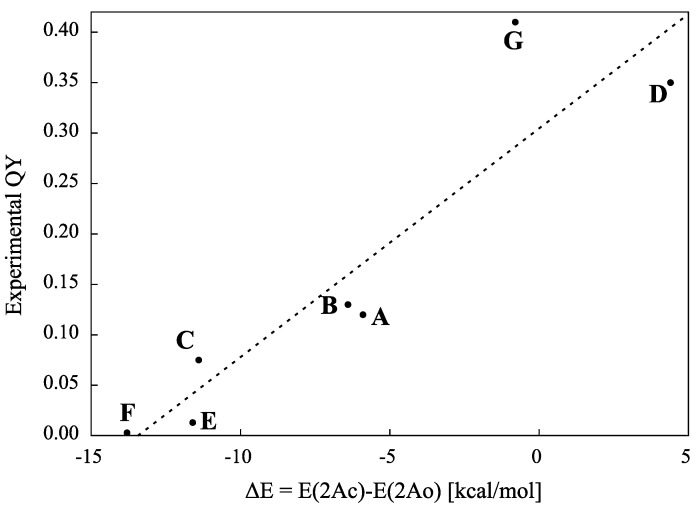
Correlation of experimental QY with calculated ∆E = E(2Ac) − E(2Ao). The C_5_F_6_ parts were truncated in the calculations, the level of which was CASSCF(10,10)/6-31G.

Given the PES for the model molecule as mentioned above, it is possible to rationalize the experimental QYs obtained for various derivatives. The experimental QYs obtained for the cycloreversion reaction 2A are shown in Figure 3. On photo-excitation of the closed-ring isomer (1Ac in Figure 1), the excited state dynamics starts on the 2A surface. In the PES profiles shown in Figure 1, the topology of the 2A excited state suggests that there is a barrier between 2Ac and 2Ao. This barrier, the 2A transition state (2A TS), obviously will determine the kinetics. Although it is very difficult to determine the transition state of the excited states for all the derivatives by current theoretical methods, a first approximation to evaluating the barrier is to compare the energy of the reactant with that of the product. Comparing E(2Ac) and E(2Ao) and then plotting the ∆E *versus* experimental QYs, we obtain the linear relation shown in Figure 4.

It is noteworthy that the electron-withdrawing substituent –CN gives the largest QY (0.41), whereas the electron-donating substituent –OCH_3_ gives the smallest (0.0029). The bond length of the reactive carbon C2-C2' at 2Ac reflects also this trend. For example, at the molecule D which is with benzothiophene substitution, the relatively large QY (0.35) value also reflects the ∆E of 4.4 kcal/mol as well as the bond length (1.6A) [8,30].

## 5. QYs for Inverse-Type DAEs

**Scheme 4:**
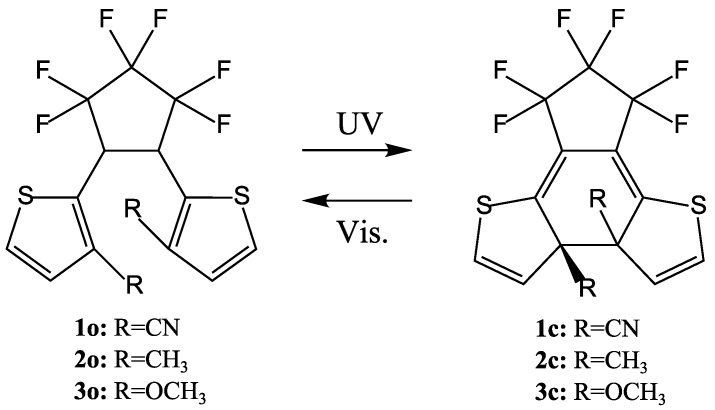
Molecules with representative substitution.

Comparing Figure 1 and Figure 2, the difference between the PES profiles is obvious. The PESs and their conical intersection must determine the QYs. It is important, however, to verify the substituent effect for both categories of DEA molecules. Although the synthesis of the inverse-type of molecules was not much reported, bis(i2-thienyl)perfluorocyclopentenes with the representative substituents –CN, –CH_3_, and –OCH_3_ (Scheme 4) were synthesized. Their spectroscopic properties in hexane solution are listed in Table 1 [35].

**Table 1:** Spectrocopic properties of bis(2-thienyl)perfluorocyclopentenes in hexane solution.

	λ_max_/nm (ε/M^−1^ cm^−1^)	Φ_oc_		λ_max_/nm (ε/M^−1^ cm^−1^)	Φ_co_
**1o**	258 (1.27 × 10^4^)	0.17 (366 nm)	**1c**	427 (9.1 × 10^3^)	0.45 (435 nm)
	289 (1.30 × 10^4^)				
	236 (1.24 × 10^4^)				
**2o**	319 (1.55 × 10^4^)	0.25 (313 nm)	**2c**	432 (8.8 × 10^3^)	0.37 (435 nm)
**3o**	327 (1.8 × 10^4^)	0.22 (366 nm)	**3c**	481 (5.8 × 10^3^)	0.25 (435 nm)

**Figure 5:**
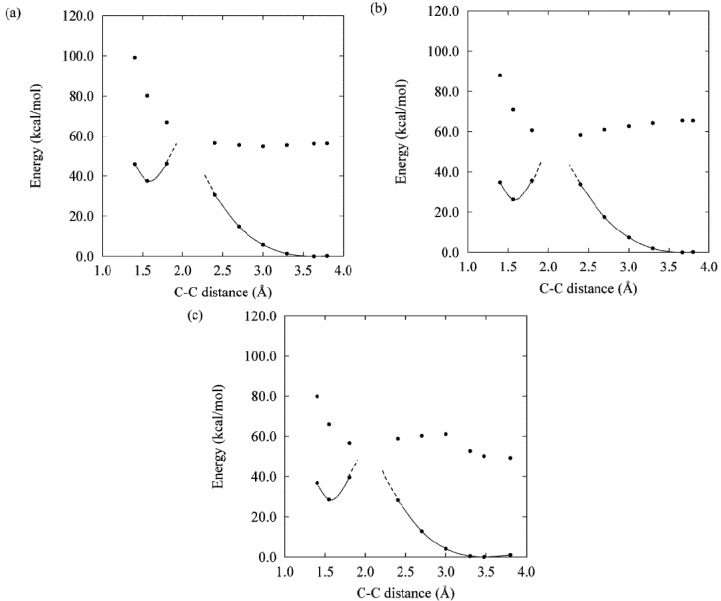
Potential energy surface of ground state (solid line with dots) and excited state (S_1_) (dots): (**a**) molecule **1**, (**b**) molecule **2**, and (**c**) molecule **3**. These values are obtained by optimization of the S_0_ and S_1_ states by fixing the distance between reactive carbon atoms.

The cycloreversion QY for the inverse-type derivative with R=OCH_3_ is remarkably large–0.25. This value is surprising when we compare it with the very small value seen for the normal-type derivative (0.0029) (see Figure 3, Figure 4), but it is understandable when we note the PES profile shown in Figure 2. The PES profiles for the inverse-type of derivative shows that for cycloreversion there is no barrier on the 2A surface; instead there is a simple downhill profile. The PES profiles for the three representative inverse-type diarylethene derivatives—bis(2-thienyl)perfluorocyclopentenes with –CN, –CH_3_, and –OCH_3_ substituents—are shown in Figure 5.

In the ground state of the closed form, the calculated distances between the reactive carbon atoms (C2, C2′) are 1.560 Å for **1**, 1.559 Å for **2**, and 1.551 Å for **3**. The longer the calculated bond length, the larger the QY of the cycloreversion reaction. The energy of the excited state of the closed-ring isomer relative to that of the ground state of the open-ring isomer is 80.1 kcal/mol for molecule **1**, 71.1 kcal/mol for molecule **2**, and 66.2 kcal/mol for molecule **3** (Figure 5). Geometries around 2.0 Å (near TS of S_0_) are not calculated because the TDDFT method is not appropriate [35]. These results suggest that the closed-ring form of **1** is the most unstable of the three closed-ring forms and that the closed-ring form of **3** is the most stable one. The PES profiles for the closed-ring to open-ring cycloreversion of molecule **1** shows a clearer downhill profile than do those for the cycloreversions of molecules **2c** and **3c**. These data indicate that the cycloreversion reaction would be smoother in **1c** than in **2c** or **3c**. These findings are consistent with the experimental cycloreversion QYs (Φco in Table 1): 0.45 for molecule **1**, 0.37 for molecule **2**, and 0.25 for molecule **3**. 

## 6. Two PES Profiles and Remaining QY-Design Problems

**Figure 6:**
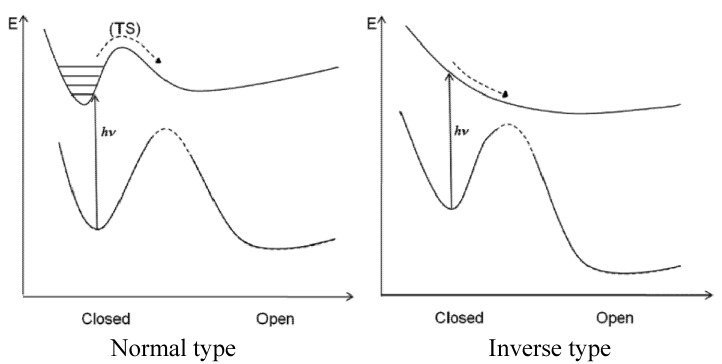
Schematic PES profiles for normal- and inverse-type DAEs.

Summarizing the analysis described above, the PES profiles for normal- and inverse-type DAEs are shown schematically in Figure 6. On the basis of various synthesized examples of normal-type DAEs and one example of an inverse-type DAE, we previously proposed a hypothetical PES for inverse-type DAEs that was different from the PES for normal-type DAEs that was based on calculations for model molecules [8]. Now that there is more experimental data consistent with the theoretical PES for inverse-type DAEs (Figure 2), it seems that the PES for a normal-type DAE has a barrier on the 2A surface and the PES for an inverse-type DAE does not. 

**Figure 7:**
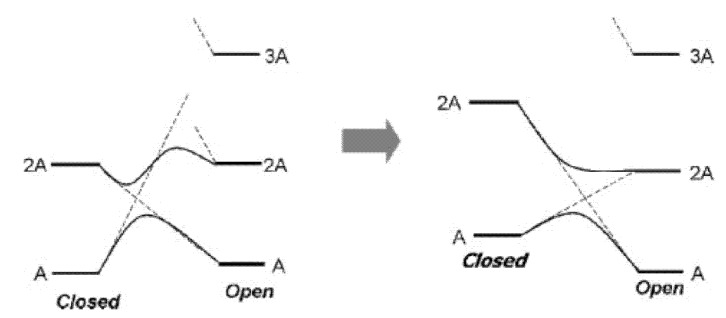
PES profile change from normal-type (left, with barrier on 2A surface) to inverse-type (right, without barrier on 2A surface).

The rationalization from diabatization has been described previously [8]. For a normal-type DAE the thermodynamic stability of the ground state of the closed isomer is almost the same as or slightly greater than that of the open form, whereas for an inverse-type DAE the closed form is less stable than the open form (Figure 7). The stability difference between the ground states of the closed and open forms thus suggests the existence or absence of the barrier on the 2A surface. The quantum yield of the transition between the closed and open forms would thus depend on the presence or absence of a barrier on the 2A potential energy surface. Since the current molecules belong to the same family of DAEs, it is still to be cautious to generalize this for other molecules. In other words, although the PES profile can provide insight for QY, at least two more essential factors are to be considered for QY determination, the location of conical intersections and quantum dynamics in excited states. 

The PES profiles thus provide a rationalization for the counterintuitive QY values for each category of DAE. We believe that this theoretical information is indispensable for future molecular design, but there are still QY-design problems open to theoretical and experimental research. Typical examples include the fact that in the future much attention will be paid to the QY estimation for complex systems, such as an azulene ring system having two excited states [36].

The following are typical questions. Is it possible to design a molecule having QY = 1.0 for both cyclization and cycloreversion [21]? In other words, are the conical intersections for cyclization and cycloreversion the same? What determines the relation of fluorescence QY and reaction QY? To what extent can the spin state be operational in photochromic reactions? What is the detailed character of higher excited sates [37]? Can multi-photon reactions be controlled? The complete answers for these questions are future subjects which will request more computer resources for theoretical works. 

The experimental measurement of QY was not an easy task, although the principle is clear and obvious. The determination in liquid solutions now became a common task and it is usually performed with conventional methods by actinometry or pump-probe techniques. As far as DAE is concerned, the first measurement is reported by Irie *et al.* [37], then the results are gathered in the review in 2000 [3]. The recent results are covered in a new book [38]. According to ultrafast laser photolysis studies, the excited state lifetimes of closed-ring forms are in the range of a few ps to a few tens of ps [39,40,41,42]. These lifetimes are two to three orders shorter than the fluorescent lifetimes of typical organic dyes. No clear correlation of excited state lifetime with cycloreversion QY is observed (see chapter 12 of ref. [38]). Ishibashi *et al.* reported for the molecule D of Figure 3 that there is temperature dependence of the excited state dynamics and QY in n-hexane solution, by contrast there is no temperature dependence in cyclization. They suggested the presence of several nonradiative decay channels which compete with the cycloreversion reaction. Also suggested by them is that these very rapid nonradiative processes and the cycloreversion reaction have activation energies different from each other [41]. These experimental results are consistent with the arguments described by current theoretical studies, although it is in the level of qualitative comparison. The next challenge for theoretical study is to understand the experimental results of multiphoton-gated cycloreversion photochromic reactions, that is, the understanding of the mechanism of nonlinear phenomena [21,38,42].

This review focused on the relation of PES and QY, therefore the review of recent development in experimental works is out of the scope. However, there are various works closely related to the current discussion [43,44,45,46,47,48,49,50,51,52,53]. Examples include recently reported interesting experimental results; the possibility to exploit DAE and a new kinetic method to determine photoreaction QYs in the visible region is reported [43,44], also a kinetic model describing the conversion of the photoactive species from both analytic and numeric solutions which is applied to measure the quantum yield of DAE-based polymers [45]. The environmental effect on the QY is also one of the most important problems to be controlled for future application. It is noteworthy that the QY measurement of the reaction in single crystal is reported; the extremely high QY value of 100% has exhibited [33]. Also noteworthy is that the QY of the single molecule measurement fluctuates as a function of Tg of the environment polymers, we have reported the study of the mechanism [19,46].

## 7. Conclusions

Molecular electronics is one of the most promising technologies in the near future, owing to the size (one molecule is in itself one quantum dot), the manipulation feasibility, and the variety [54]. The photochromic DAEs are one of the most important candidate molecule systems for this new technology. In an attempt to contribute via theoretical study, we presented the relation between QY and PES profile. The counterintuitive property QY is thus explained for two categories of DAE derivatives. The profile of the excited-state PES explains the QY trend of DAEs with representative substituents such as CN, CH_3_, and OCH_3_. A natural extension for the theoretical study of QY is also described.
